# Development of a Redox-Polymer-Based Electrochemical Glucose Biosensor Suitable for Integration in Microfluidic 3D Cell Culture Systems

**DOI:** 10.3390/bios13060582

**Published:** 2023-05-27

**Authors:** L. Navarro-Nateras, Jancarlo Diaz-Gonzalez, Diana Aguas-Chantes, Lucy L. Coria-Oriundo, Fernando Battaglini, José Luis Ventura-Gallegos, Alejandro Zentella-Dehesa, Goldie Oza, L. G. Arriaga, Jannu R. Casanova-Moreno

**Affiliations:** 1 Centro de Investigación y Desarrollo Tecnológico en Electroquímica, Pedro Escobedo 76703, Querétaro, Mexico; lnavarro@cideteq.mx (L.N.-N.); jgonzalez@cideteq.mx (J.D.-G.); daguas@cideteq.mx (D.A.-C.);; 2 Instituto de Química Física de los Materiales, Medio Ambiente y Energía, CONICET—Departamento de Química Inorgánica, Analítica y Química Física, Facultad de Ciencias Exactas y Naturales, Universidad de Buenos Aires, Buenos Aires C1428EGA, Argentina; coria@qi.fcen.uba.ar (L.L.C.-O.); battagli@qi.fcen.uba.ar (F.B.); 3 Departamento de Medicina Genómica y Toxicología Ambiental, Instituto de Investigaciones Biomédicas, Universidad Nacional Autónoma de México, Ciudad de México 04510, Mexico; 4 Unidad de Bioquímica, Instituto Nacional de Ciencias Médicas y Nutrición Salvador Zubirán, Ciudad de México 14080, Mexico

**Keywords:** glucose electrochemical biosensor, glucose oxidase, cross-linking, 3D cell culture, on-chip evaluation, glutaraldehyde, EGDGE

## Abstract

The inclusion of online, in situ biosensors in microfluidic cell cultures is important to monitor and characterize a physiologically mimicking environment. This work presents the performance of second-generation electrochemical enzymatic biosensors to detect glucose in cell culture media. Glutaraldehyde and ethylene glycol diglycidyl ether (EGDGE) were tested as cross-linkers to immobilize glucose oxidase and an osmium-modified redox polymer on the surface of carbon electrodes. Tests employing screen printed electrodes showed adequate performance in a Roswell Park Memorial Institute (RPMI-1640) media spiked with fetal bovine serum (FBS). Comparable first-generation sensors were shown to be heavily affected by complex biological media. This difference is explained in terms of the respective charge transfer mechanisms. Under the tested conditions, electron hopping between Os redox centers was less vulnerable than H_2_O_2_ diffusion to biofouling by the substances present in the cell culture matrix. By employing pencil leads as electrodes, the incorporation of these electrodes in a polydimethylsiloxane (PDMS) microfluidic channel was achieved simply and at a low cost. Under flow conditions, electrodes fabricated using EGDGE presented the best performance with a limit of detection of 0.5 mM, a linear range up to 10 mM, and a sensitivity of 4.69 μA mM^−1^ cm^−2^.

## 1. Introduction

Recent advances in 3D culturing design and technology have improved cell research. Microfluidics play an important role in enabling better control of cell cultures to resemble in vivo conditions. In them, it is possible to evaluate the response to stimuli, metabolic parameters, cell interactions, and treatments [1]. However, developing a biological model requires a strong framework to grow cells in ideal conditions and to examine cell parameters. To ensure the cultures represent the physiology of the desired tissue, not only the spatial arrangement of the cells needs to be characterized. The environment around the cells must also be monitored since it affects cell viability, growth, differentiation, and function [2]. Currently, most of the analysis is performed outside the chip, losing important information, resources, and time. Therefore, it is crucial to evaluate, in real-time, cellular parameters that allow us to control the conditions inside the chip and to better understand disease behavior and the potential of novel drugs. Thus, it is essential to develop online, in situ sensors with high sensitivity and stability, as well as to develop sensors resistant to the fouling caused by the continuous exposure to the complex cell culture medium [3].

Ideally, all parameter measurements are combined to discuss the proper biological functionality of the model under different conditions. Glucose determination is important to monitor metabolic activity in 3D cell cultures and organ-on-a-chip models, since it can be related to mitochondrial function [2,3,4] and cell proliferation [5,6,7]. Quantification of glucose in biological fluids is a well-established industry for monitoring diabetic patients. Although some colorimetric and fluorescence methods have been reported [8], commercial glucose meters mostly rely on electrochemical biosensors using glucose oxidase (GOx) or glucose dehydrogenase (GDH) immobilized on electrode surfaces [9]. On the other hand, in 3D cell cultures, cellular response to certain stimuli is frequently analyzed with optical and fluorescence microscopy using stains and labels. Nonetheless, some drawbacks of these methods are that labels can interact nonspecifically with the cells and substances under testing, generating interferences or quenching of the luminescence [10]. Other limitations of optical sensors include biofouling when exposed to cell culture media, photodegradation of sensor dyes limiting long-term stability, and the inability to achieve noninvasive detection [10,11]. These characteristics hamper their integration in complex platforms for in-line analysis in continuous evaluations, most of the reports being for 2D cell cultures [12,13,14,15,16,17]. Since integrated sensors need to demonstrate long-term stability, reliability, and robustness to meet the high standards required in clinical diagnosis, the development of new methodologies is becoming increasingly important. Electrochemical sensors are attractive for their low costs, quick response, and capacity to evaluate different analytes simultaneously [6,18,19,20,21].

Enzymatic electrochemical biosensors are usually classified in so-called generations based on the charge transfer mechanism from the enzyme active center to the electrode surface [22]. In first-generation enzymatic electrochemical glucose biosensors, the production of hydrogen peroxide by GOx is quantified amperometrically. Some of the main limitations of these sensors are the signal dependence on the oxygen concentration and hydrogen peroxide accumulation for continuous electrochemical monitoring, producing adverse effects on cells [3]. In second-generation enzymatic electrochemical biosensors, redox-active agents replace natural electron acceptors and are used as mediators, allowing the flow of electrons between the redox center of the enzyme and the electrode surface (typically a noble metal or carbon). Using this strategy, it is possible to work with lower potentials than in first-generation sensors, decreasing interferences of other electroactive species [23]. It is important to immobilize the mediators closely to the enzymes and the electrode surface to allow electron transfer. The immobilization method will influence the stability, linear range, selectivity, and response time of the system [24]. In cross-linking, the reactants form covalent bonds with enzyme surface groups. Most of the known cross-linkers, such as ethylene glycol diglycidyl ether (EGDGE) and glutaraldehyde (GA), have epoxy or aldehyde groups in their ends [25]. These groups can react with amine, carboxyl, and hydroxyl groups. The enzymatic cross-linking takes place with lysine ε-amine groups present in the structure of the GOx, and the amine groups of a polymer such as polyethyleneimine or polyallylamine. In this way, networks with high complexity can be obtained to improve the performance [24].

Besides the benefits listed above, electrochemical sensors are easily miniaturized, making them a prime candidate for integration in organ-on-a-chip platforms [2,18,19,20]. Furthermore, since the enzymes are usually immobilized on the electrode surface, electrochemical sensors are more efficient for flow applications than colorimetric or fluorescence sensors in which the enzyme is typically in solution. Culture media contain complex mixtures of compounds and, consequently, represent challenging environments for enzymatic glucose biosensors. Complications such as enzyme stability and activity limit the sensor’s dynamic range and operational stability, as well as other aspects of performance [3,7]. As a result, the integration level has varied greatly; most reports are off-chip to minimize negative effects on the electrodes caused by the culture medium complexity. Bavli et al., for example, used a separate sensing module in which commercial first-generation biosensors on screen-printed electrodes (SPEs) were sealed using an o-ring. Samples were evaluated continuously for 24 h in a low-glucose modified eagle medium with 10% FCS, and 1% penicillin-streptomycin [4]. Tang et al., used a complex second-generation glucose biosensor on graphite SPEs modified with carbon black-prussian blue as a mediator layer and a Nafion membrane. The evaluations were carried out in Dulbecco’s Modified Eagle (DMEM) medium (NIH 3T3 fibroblasts were cultured in 5.5 mM glucose DMEM medium, supplemented with 10% fetal bovine serum (FBS) and 1% penicillin-streptomycin). Nonetheless, to minimize the complex matrix effect on the biosensor performance, the analysis was carried out off-chip, and the medium was diluted 1:4 (v:v) in a phosphate buffer (PB) [7]. With a similar approach, Tehuer et al., designed a first-generation biosensor on carbon electrodes with a chitosan membrane. Lactate was measured in (Roswell Park Memorial Institute) RPMI, Luria-Bertani and CgXII media. However, the biosensor was designed for single-use and samples required a pre-treatment before evaluations. Stability was analyzed off-chip, taking samples every hour in a 24 h period [26]. Another example by Madhurantakam et al. reported a third-generation biosensor on a hybrid carbon nanotube–graphene electrode. Glucose was measured just at discrete times for 12 h in a DMEM medium (MiaPaCa-2 cells cultured in DMEM medium supplemented with 10% FBS and 1% penicillin-streptomycin). However, samples were evaluated off-chip and just in a few time points to avoid fouling [6]. On the other hand, Dornhof et al., reported a complex first-generation biosensor on platinum electrodes with a membrane to improve the performance for glucose analysis. The biosensor was placed directly in a mammary epithelial basal medium (not in direct contact with cells) for 7 days, reporting a stable performance without fully describing the biosensor behavior parameters [18]. In a similar way, Ma et al. designed a complex second-generation biosensor to evaluate glucose concentrations in a high glucose DMEM medium with 10% FBS in which human lung cancer PC9 cells were cultured. However, performance parameters are not mentioned [5]. In summary, glucose detection in most of the analyses in complex cell culture platforms are carried out off-chip, increasing design complexity; in these cases, biosensors are not in direct contact with cells, and samples are not continuously monitored and usually need a pretreatment. On the other hand, methodologies for electrode modification are frequently very complex and most of the time involve the use of membranes. Therefore, there is a need for friendly methodologies to monitor microfluidic cell cultures in real-time, close or even in direct contact with the cells.

Carbon, in several of its forms, is one of the most common electrode materials because of its good conductivity and low cost, among other qualities [27]. However, the incorporation of carbon electrodes in channel-based microfluidic systems is less straightforward than integrating metal electrodes, which are routinely deposited by electron beam evaporation [28,29]. Ingenious strategies have included photoresist pyrolysis [30] and molding of carbon materials inside polydimethylsiloxane (PDMS) structures [31,32]. As an alternative, pencil leads (sometimes referred to as pencil graphite electrodes, PGE) have been used as electrodes for a variety of analytical applications [33]. They have great potential because they are easy to manufacture, easy to modify, present high stability, good sensitivity, reproducibility, and resistance to surface passivation [34,35,36]. Nowadays, they are widely used for the determination of a large variety of compounds in different samples, ranging from simple matrices to complex biological fluids (urine, plasma, human serum, DNA, beverages, etc.) [33,34,35,37]. Furthermore, they can be acquired commercially with nominal diameters down to 0.2 mm at a low cost. Because of their dimensions, these electrodes have been incorporated in microfluidic devices [34,35]. Witkowska et al., for example, reported a microfluidic device with carbon pencil leads incorporated for p-Nitrophenol and dopamine detection [34]. Islam et al. designed a sensor for free residual chlorine using pencil graphite leads as working electrodes [38]. Senel et al. designed a microfluidic platform with integrated PGEs for clozapine detection [39]. Torrinha et al. reported a microfluidic platform with an embedded pencil graphite electrode biosensor for glucose detection. However, the PGE/graphene/MWCNT/GOx/GA/Nafion biosensor requires a complex methodology and uses a mediator in solution (2 mM benzoquinone) [36]. This could present a problem in complex biological matrices since it could drastically change the biosensor performance or results detrimental for cultured cells.

In this study, carbon pencil leads are evaluated as a low-cost option for glucose monitoring in cell culture conditions in a microfluidic device. Moreover, the performance of GA and EGDGE as cross-linkers for second-generation electrochemical biosensor design is evaluated. Glucose monitoring is analyzed directly in cell culture medium on-chip to determine the biosensor behavior under normal cell culture conditions for future integration in a 3D cell culture system.

## 2. Materials and Methods

### 2.1. Reagents and Solutions

GA (50 wt % in water), GOx from *Aspergillus niger* (100,000–250,000 U/g), glucose (β-D-glucose ≥ 99.5%), branched polyethyleneimine (BPEI, Mw ~25 KDa), monobasic potassium phosphate (KH_2_PO_4_ ≥ 99.0%), dibasic potassium phosphate (K_2_HPO_4_ ≥ 99.0%), RPMI-1640 medium (with L-glutamine, without phenol red and sodium bicarbonate), FBS, and DMEM low glucose medium (1000 mg/L = 5.5 mM, with L-glutamine, sodium bicarbonate, and phenol red) were purchased from Sigma Aldrich (Saint Louis, USA). DMEM high glucose medium (4500 mg/L = 25 mM, with L-glutamine, sodium bicarbonate, and phenol red) was acquired from Gibco (New York, USA). Polydimethylsiloxane (PDMS) Sylgard 184 was acquired from Dow (Midland, USA). EGDGE (100 wt %) was acquired from Polysciences, Inc (Niles, USA). Os(bpy)_2_Cl(pyCOH) covalently bound to BPEI (OsBPEI) was synthesized according to a procedure previously reported [40]. The solutions were prepared with ultrapure water with a resistivity of 18.2 M Ω·cm from a Merck Millipore purification system (simplicity UV) with a 0.22 μm filter, and stored at 4 °C. Additionally, a high temperature stereolithography resin was acquired from eSUN (Shenzhen, China).

### 2.2. Electrochemical Cell Design

First evaluations were made off-chip in two different electrochemical cells. A drop-based electrochemical cell for 50 μL samples was fabricated in-house by screen printing electrodes over an insulating Mylar substrate [40]. The design consisted of a circular working electrode (WE) with an area (A) of 0.086 ± 0.006 cm^2^ partially surrounded by a larger counter electrode (CE) and a smaller pseudo-reference electrode (RE) (Figure S1). All the inks used were purchased from Gwent (Torfaen, UK). First, silver ink tracks were deposited on the Mylar to improve electrical conductivity. A layer of carbon ink was deposited on top of the silver tracks to form the WE and CE electrodes. This deposit was wider than the silver tracks to make sure no silver was exposed to the working solution. A similar process was carried out to deposit the reference electrode but using a mixture of carbon and silver inks spiked with silver chloride prepared in house. Finally, an insulating layer was deposited to outline the electrochemical cell, avoiding contact of the electrolyte solution with the connection pads. For the second design, five different brands of carbon pencil leads with a 500 μm nominal diameter were evaluated in a 5 mL beaker with 4 mL samples. Pencil leads were used as WE and CE, and a Ag|AgCl wire as a RE.

### 2.3. Electrode Modification

Initially, SPEs and carbon pencil leads were cleaned electrochemically by cyclic voltammetry (CV) in 0.1 M pH 7.4 phosphate buffer (PB). Subsequently, for second-generation enzymatic electrodes (OsBPEI/GOx/GA or OsBPEI/GOx/EGDGE), fresh solutions of 5 mg/mL (31.2 µM) GOx in 0.1 M pH 7.4 PB and 0.48 mM OsBPEI (osmium:amine molar ratio of 1:12) were mixed. Then, to evaluate the cross-linker behavior and select the most effective composition, GA or EGDGE were spiked and mixed to achieve concentrations of 3.33, 17.5, or 33.3 mM. For the fabrication of first-generation enzymatic electrodes (BPEI/GOx/GA or BPEI/GOx/EGDGE), fresh solutions of 5 mg/mL GOx in 0.1 M pH 7.4 PB and 10 mg/mL (0.4 mM) BPEI in water were mixed. Next, GA or EGDGE was spiked and mixed to a final concentration of 33.3 M. Finally, 3 μL of the mixture were deposited by drop-casting on the carbon surfaces. The hydrogels were allowed to dry for 60 min at 30 °C and rinsed with ultrapure water to remove any excess.

### 2.4. Electrochemical Analysis

First- and second-generation enzymatic biosensors were evaluated and compared. Initially, CV was performed to compare differences in peak current and peak potential between electrodes and to identify the cathodic and anodic peaks of the Os(II)/Os(III) redox pair in second-generation electrodes, or the H_2_O_2_ oxidation potential in first-generation electrodes. The experiments were carried out in 0.1 M pH 7.4 PB, with a scan rate of 100 mV/s, applying potentials from 0.2 to 0.8 V and from −0.1 to 0.5 V vs. Ag|AgCl for first- and second-generation biosensors, respectively. Once the anodic peak or the oxidation potential was determined, we performed chronoamperometry (CA) to evaluate glucose detection. Calibration curves in 0.1 M pH 7.4 PB were obtained by plotting the current at 2 min vs. glucose concentration in the 0–100 mM range. The experiments were performed in triplicate.

To evaluate the performance of the electrochemical biosensor in a relevant biological matrix, different concentrations of glucose between 0–100 mM were prepared in RPMI-1640 cell culture medium. Additionally, five samples of RPMI-1640 with a glucose concentration of 5 mM were spiked with different percentages (0, 2.5, 5, 7.5, and 10%) of FBS to determine whether the electrochemical biosensor can work in a more complex culture medium without interferences. Moreover, modified carbon pencil lead electrodes were also evaluated in RPMI-1640 and DMEM culture medium with a high (25 mM) and low (5.5 mM) glucose concentration.

### 2.5. Imaging

Bright-field color images of the modified pencil lead electrodes were acquired using a Distamax K2 long distance microscope (Centennial, USA) coupled to a Canon EOS Rebel T5 DSLR camera (Melville, USA). Fluorescence images were obtained using a Nikon Ti-U inverted microscope (Melville, USA) equipped with the Chroma 49002 filter set (450–490 nm exc., 500–550 nm emi. and 495 nm dichroic, Bellows Falls, VT, USA) and registered in a monochromatic Qimaging optiMOS camera (Surrey, Canada) through a 4× objective. Fluorescence images were acquired at different focal planes and stitched together using the Extended Depth of Field plugin [41] for ImageJ (version 1.52a) [42]. Scanning electron microscopy (SEM) and energy dispersive X-ray (EDX) mapping were performed in a Zeiss EVO 15 Scanning Electron Microscope (Jena, Germany). Samples were conductive enough that they did not require Au coating to avoid charging.

### 2.6. Microfluidic Channel Fabrication

**Figure 1:**
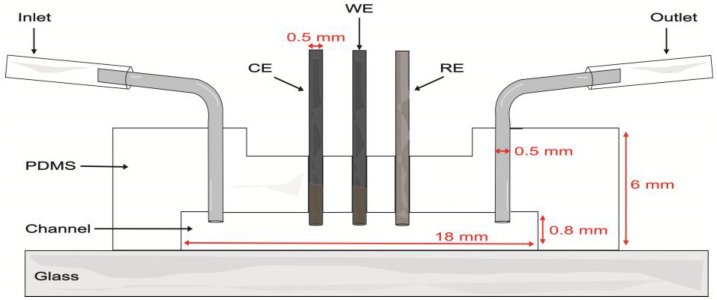
Schematic of the microfluidic channel with the integrated carbon pencil leads as working (WE) and counter (CE) electrodes and a Ag|AgCl wire as pseudo-reference electrode (RE).

Proof-of-concept evaluations were performed in a single-channel model with a length of 18 mm, width of 1.5 mm, and depth of 0.8 mm. The soft lithography mold was created by 3D printing (Elegoo Mars 3 printer, Shenzhen, China) with eSUN High Temp Resin. The mold was filled with a PDMS base and curing agent at a weight ratio of 10:1 and degassed under vacuum for 45 min to remove unwanted air bubbles. The PDMS was cured in an oven at 60 °C for 4 h. After demolding, inlet and outlet holes were made in the PDMS with a biopsy punch (0.5 mm diameter). Three additional holes were made along the channel to introduce the WE, CE, and RE electrodes. Then, the PDMS layer and a microscope glass slide were bonded after undergoing air plasma treatment for 75 s. Metal connectors were inserted into the inlets and outlets of the channel (Figure 1). To clean the device, 0.1 M pH 7.4 PB was passed through the channel for 5 min. A nominal flow rate of 100 μL/min was used in evaluations within the microfluidic channel.

### 2.7. Data Analysis

Chronoamperometric data were analyzed to understand the behavior of the different hydrogel compositions. Being catalytic biosensors, enzymatic sensors are characterized in terms of their Michaelis constant (K_m_) and maximum rate (V_max_) values. In electrochemical enzymatic biosensors, electrical current measurements instead of enzymatic reaction rates yield “apparent” Michaelis–Menten constants (K_m_^app^) and maximum currents (i_max_), which are commonly used to assess sensor performance [43]. These two parameters were calculated from non-linear fitting of the data to a hyperbolic function representing the Michaelis–Menten model. The lowest current value (corresponding to the blank except in first-generation experiments) was subtracted in each data set analysis. To facilitate comparisons between the different types of electrodes employed in this work, area-normalized values of maximum current density (j_max_) are reported instead of i_max_. To analyze reproducibility, the experiments were made in three different carbon SPEs or pencil lead electrodes. The limit of detection (LOD) and limit of quantification (LOQ) are defined as the lowest analyte concentration to be reliably detected and quantitatively determined with accuracy and precision, respectively. They were calculated using the following equations:

(1) $$LOD=\frac{3s}{m}$$

(2) $$LOQ=\frac{10s}{m}$$

where s is the standard deviation of the intercept and m is the slope of the calibration curve (*n* = 3) [44]. The linear range was considered from zero to the highest measured concentration which yielded a linear regression where the coefficient of determination (R^2^) is equal or greater than 0.98. The sensitivity was calculated as the slope of the regression fit in the linear range. Moreover, three calibration curves were made in the same electrode to evaluate the stability of hydrogels. The remaining percentage of the initial value of i_max_ after the third calibration curve was considered as a measure of the sensor stability.

## 3. Results and Discussion

### 3.1. Second-Generation Electrodes in Carbon SPEs

#### 3.1.1. Cross-Linker Evaluation

**Figure 2:**
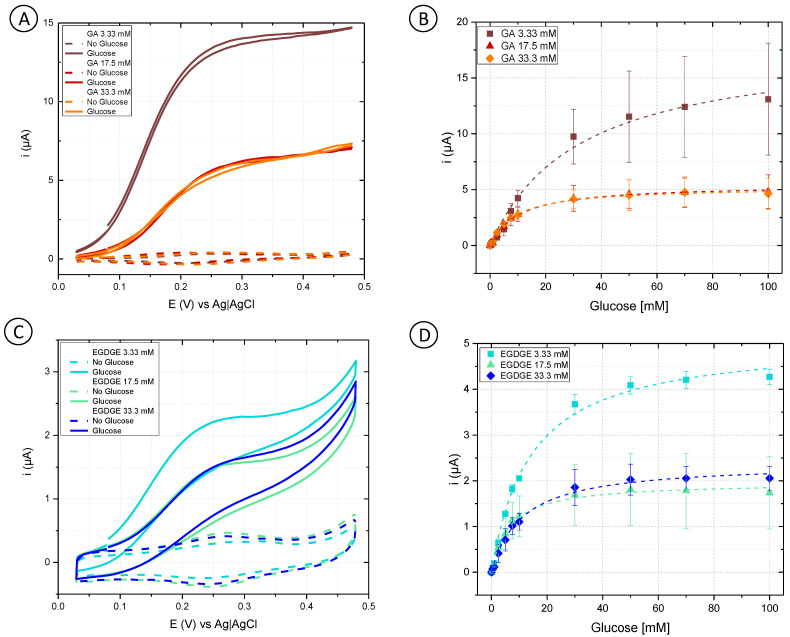
(**A**,**C**) Cyclic voltammograms of hydrogels containing glucose oxidase (GOx) cross-linked to branched polyethyleneimine modified with Os(bpy)_2_Cl(pyCOH) (OsBPEI) using either glutaraldehyde (GA) or ethylene glycol diglycidyl ether (EGDGE). (**A**) OsBPEI/GOx/GA and (**C**) OsBPEI/GOx/EGDGE hydrogels with different cross-linker concentrations in 0.1 M pH 7.4 phosphate buffer (PB), with 0 mM (dotted lines) and 100 mM (continuous lines) glucose. Scan rate: 2 mV/s. (**B**,**D**) Calibration curves for 0–100 mM glucose in0.1 M pH 7.4 PB, based on chronoamperometric evaluations using (**B**) OsBPEI/GOx/GA and (**D**) OsBPEI/GOx/EGDGE hydrogels with different cross-linker concentrations. Error bars represent the standard deviation between three independent electrodes. Dashed lines represent the corresponding curves employing the apparent Michaelis–Menten constant (K_m_^app^) and maximum current (i_max_) calculated through non-linear fitting.

Initially, cyclic voltammetry was performed in a phosphate buffer to analyze the behavior of different concentrations of GA and EGDGE (Figure 2A,C). In the absence of glucose, the anodic and cathodic peaks for the Os(II)/Os(III) can be observed in the 0.15–0.3 V range depending on the system (Figure S2). Experiments at different scan rates yielded a linear dependence of the current with respect to the scan rate, indicating a thin layer regime (Figure S3). When glucose was present at 100 mM concentration, the system using GA as a cross-linker presented a sigmoidal wave consistent with a catalytic regeneration of the reagent [45,46]. EGDGE-based hydrogels presented the same catalytic increase in the current but to a lesser extent, with limiting currents about seven times smaller. This lower current when using EGDGE as cross-linker instead of GA has been previously reported by our group [24]. In addition, chronoamperometry was carried out to evaluate the effectiveness of glucose detection. The potential for these experiments was chosen so as to be slightly higher than the anodic peak of the CVs in the absence of glucose. These calibration curves were analyzed in terms of Michaelis–Menten enzyme kinetics (Table S1). Among the GA-based hydrogels, the highest j_max_ (208 ± 13 µA/cm^2^) was observed when using the lowest concentration (3.33 mM) of cross-linker (Figure 2B). This can probably be explained by the looser structure caused by the lower cross-linking density, which improves glucose diffusion compared to higher cross-linker concentrations. The electrode prepared with 33.3 mM GA showed the lowest j_max_ (60.9 ± 1.0 µA/cm^2^); nonetheless, it yielded the lowest LOD (0.12 mM) and LOQ (0.39 mM), and the highest stability of the three evaluated concentrations (Table 1 and Table S2). Based on the results, OsBPEI/GOx/GA 33.3 mM was selected for the following evaluations. As in the cyclic voltammograms, EGDGE-based hydrogels presented lower currents. The highest j_max_ corresponded to the 3.33 mM cross-linker concentration and was only 58.9 ± 1.5 µA/cm^2^ (Figure 2D). These electrodes lost almost all of their functionality after the second calibration curve. Regardless, the most stable OsBPEI/GOx/EGDGE composition (33.3 mM) was evaluated in the following experiments.

Table S1 summarizes the results of fitting the calibration curves to the Michaelis–Menten equation, which describes the activity of most enzymes. In electrochemical biosensors, a modified version of the equation relates the current–concentration relationship to yield the approximate value of K_m_^app^ and i_max_. Values of K_m_^app^ between 4.6–14.8 mM were calculated from the evaluations of most of the hydrogel combinations. These values are lower than the 26 mM K_m_ reported for the free enzyme [47]. K_m_ is frequently used as an indicator of a substrate’s binding affinity for the substrate (although determination of a truly thermodynamic dissociation constant would require non-kinetic measurements [48]). In enzymatic hydrogels, however, the difference between K_m_ and K_m_^app^ can also arise from substrate preconcentration in the hydrogel as a result of partition equilibrium [47]. An exception to this K_m_^app^ range were the electrodes prepared with 3.33 mM GA. In this case, values of K_m_^app^ between 19.9–43.1 mM were observed. However, the quality of the fitting was also lower in these electrodes, perhaps indicating further complexities related to the inferior performance of this system.

#### 3.1.2. Evaluation of Hydrogels in Culture Medium

One of the main objectives of this work is to integrate the enzymatic electrochemical biosensors in a platform that allows emulating a physiological environment that represents the tumor microenvironment in breast cancer. For this purpose, it is necessary to analyze the behavior of the biosensor with the growth conditions needed for cell culture. RPMI-1640 medium has been found suitable for a variety of mammalian cells, including MCF-7 [49,50,51,52], RAW 264.7 [53,54,55], and EAhy 926 [56,57,58]. The medium contains the reducing agent glutathione and high concentrations of vitamins such as B12, inositol, choline, and *p*-aminobenzoic acid (PABA), including certain vitamins not found in the most common culture mediums, Eagle’s Minimal Essential Medium (EMEM) or DMEM. Since RPMI-1640 contains no proteins, lipids, or growth factors, it is commonly supplemented with FBS [49,50,51,52,53,54,55,56,57,58] which has the growth, proliferation, and adhesion factors needed for most human and animal cells. It must be kept in mind, however, that its complete composition is still unknown [59].

**Figure 3:**
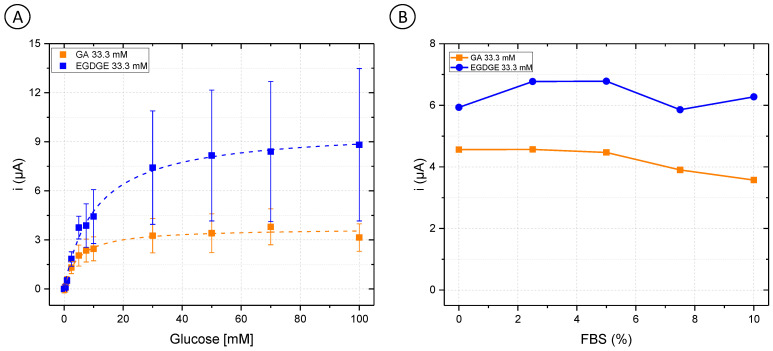
(**A**) Glucose calibration curves (0–100 mM) in Roswell Park Memorial Institute (RPMI-1640) culture medium with OsBPEI/GOx/GA or OsBPEI/GOx/EGDGE 33.3 mM hydrogels. Error bars represent the standard deviation of three independent electrodes. Dashed lines represent the corresponding curves employing the K_m_^app^ and i_max_ calculated through non-linear fitting. (**B**) Evaluation of OsBPEI/GOx/GA or OsBPEI/GOx/EGDGE 33.3 mM hydrogels with concentrations of fetal bovine serum (FBS) between 0–10% in RPMI-1640 culture medium containing 5 mM glucose.

First, OsBPEI/GOx/GA 33.3 mM and OsBPEI/GOx/EGDGE 33.3 mM were analyzed in RPMI-1640 culture medium with glucose concentrations between 0–100 mM (Figure 3A). GA-based hydrogels showed a decrease of 29.3% in their j_max_ from 60.9 ± 1.0 to 43.0 ± 1.6 µA/cm^2^ (Table S1). As well, their analytical performance was affected (Table 1), as evidenced by increased LOD and LOQ values when analyzed directly in a complex medium. Interestingly, the opposite effect was observed for EGDGE-based hydrogels, as an improvement in its behavior was observed. The value of j_max_ changed from 25.4 ± 0.7 to 113 ± 3 µA/cm^2^, and the stability increased by 43.8%. To analyze the enzymatic electrochemical biosensors in appropriate cell culture conditions, different FBS concentrations in a 5 mM glucose RPMI-1640 medium were evaluated. Both OsBPEI/GOx/GA 33.3 mM and OsBPEI/GOx/EGDGE 33.3 mM electrodes presented a relatively stable signal even in this highly complex media (Figure 3B), therefore suggesting their suitability to measure biologically relevant glucose concentrations in cell cultures.

**Figure 4:**
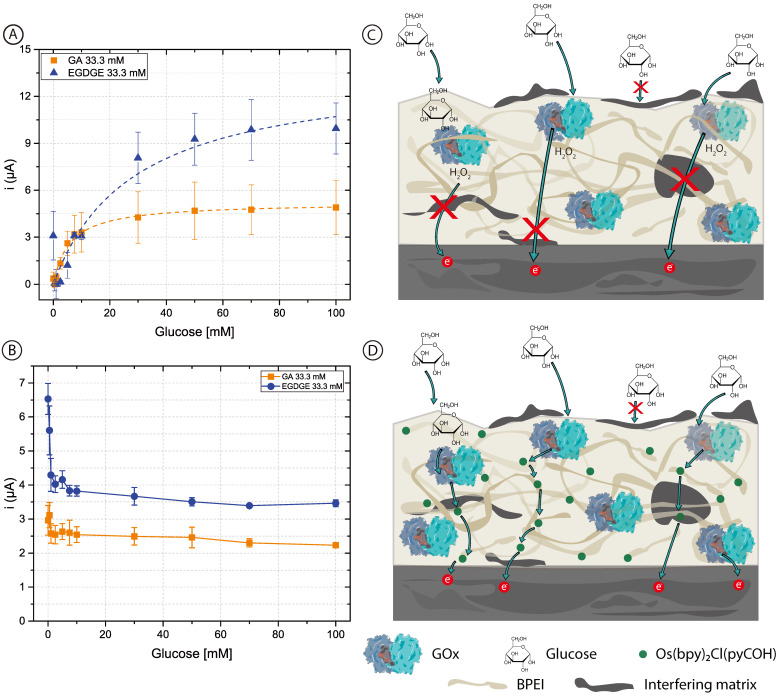
(**A**,**B**) Calibration curves of 0–100 mM glucose in (**A**) 0.1 M pH 7.4 PB and (**B**) RPMI-1640 culture medium, based on chronoamperometric evaluations employing BPEI/GOx/GA or BPEI/GOx/EGDGE first-generation biosensors. Error bars represent the standard deviation of three independent electrodes. Dashed lines represent the corresponding curves employing the K_m_^app^ and i_max_ calculated through non-linear fitting. The first point (blank) in the EGDGE series in (**A**) was not considered for the fitting. (**C**,**D**) Schematics of the effects of adsorption and absorption of media components on the charge transfer mechanisms of first- (**C**) and second- (**D**) generation biosensors.

To assess whether the added complexity in designing and fabricating a second-generation biosensor was worth it, first-generation electrodes were prepared using unmodified BPEI which does not contain mediator molecules. In this case, chronoamperograms reflect the oxidation current of the H_2_O_2_ produced by the GOx. Figure 4 shows the calibration curves with concentrations of glucose between 0–100 mM in 0.1 M pH 7.4 PB (Figure 4A) and RPMI-1640 culture medium (Figure 4B). The curves for the first-generation sensors in PB are similar to those of the second-generation sensors (Figure 2) and clearly resemble enzyme kinetics (Table 1 and Table S1). When both BPEI/GOx/GA and BPEI/GOx/EGDGE first-generation sensors were exposed to cell culture media, however, there was no increase in current in the presence of glucose (Figure 4B). Rather, an initial decrease in signal yields constant values independent of the glucose concentration. This suggests the interference from an electroactive molecule. Since higher potentials are needed to oxidize H_2_O_2_, glucose readings can present interference caused by certain substances such as glutathione, ascorbic acid, uric acid, etc., which may become co-oxidized [60]. Furthermore, any substance that absorbs in the hydrogel can potentially reduce the diffusion of glucose to the immobilized enzymes. The observed response for second-generation sensors (Figure 3A), however, indicates that at least some glucose is being oxidized by GOx even in cell culture media. In first-generation sensors, which rely on the diffusion of H_2_O_2_ to the electrode surface, non-specific adsorption of contaminants on the electrode surface can partially or totally block the access of H_2_O_2_ to be oxidized (Figure 4C). In contrast, second-generation biosensors rely on electron transport through a network of nearby mediator molecules through self-exchange redox reactions. While some of this electron transfer takes place by collisions between the redox moieties, hopping has been shown to be an important contributing mechanism [61]. This process can take place over distances larger than a nanometer [62,63]. Therefore, even if organic components get absorbed in the gel structure or adsorbed at the electrode surface, hopping can still take place between the redox moieties immobilized along with the polymer (Figure 4D). This could explain why it was not possible to have a glucose response in the first-generation electrodes in RPMI-1640 culture medium. Furthermore, it suggests that a hopping-based sensor can be more resistant to complex biological media than a diffusion-based one.

#### 3.1.3. Carbon Pencil Lead Electrodes

**Figure 5:**
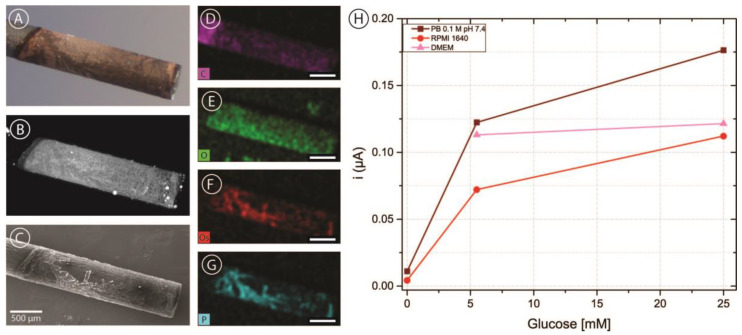
(**A**–**C**) Bright-field (**A**) fluorescence (**B**) and scanning electron microscopy (**C**) images of a Foray HB pencil lead electrode with a deposit of OsBPEI/GOx/GA hydrogel. (**D**–**G**) Energy dispersive X-ray (EDX) mapping images of the same electrode corresponding to carbon (**D**), oxygen (**E**), osmium (**F**), and phosphorus (**G**). All scale bars are 500 μm in length. (**H**) Chronoamperometric evaluations of the modified electrode in 0.1 M pH 7.4 PB, RPMI-1640 culture medium, and DMEM culture medium with glucose concentrations between 0–25 mM.

In order to incorporate carbon electrodes into the microfluidic channel, pencil lead electrodes were chosen as a simple and cost-effective approach. As described above, five brands of pencil leads were modified with OsBPEI/GOx/GA to evaluate their suitability as electrodes in our intended application. Figure 5A shows a representative bright-field microscopy image of a pencil lead electrode modified with OsBPEI/GOx/GA. It can be observed that the deposit extends approximately 2 mm from the tip, yielding an approximate geometric area of 0.071 cm^2^. The hydrogel layer can be better observed by taking advantage of the fluorescent nature of BPEI when cross-linked with aldehydes [64]. It can be observed (Figure 5B) that the deposited layer is relatively homogeneous, with some hot-spots and some darker structured regions. Scanning electron microscopy images (Figure 5C) show that these regions do have deposited material rather than being empty. Energy dispersive X-ray (EDX) mapping (Figure 5D–G) reveals that these regions present a high content of osmium and phosphorus. Osmium presence is expected from the redox polymer, while phosphate arises from the buffer solution employed through the immobilization and analysis. Oxygen is present more homogeneously throughout the whole deposit and arises from both the enzyme and the phosphate ions. Finally, the carbon signal appears decreased in the modified region compared with the bare pencil lead, as expected. After modification with OsBPEI/GOx/GA, these electrodes were evaluated off-chip with 0–100 mM glucose in 0.1 M pH 7.4 PB (Figure S4). To assess whether it was possible to measure glucose in culture media, a quick test was carried out in the absence of glucose as well as in low (5.5 mM) and high (25 mM) glucose in 0.1 M pH 7.4 PB, and RPMI-1640 medium. Low and high glucose measurements were also performed in DMEM medium (Figure 5C). No experiments were performed in DMEM in the absence of glucose as we did not have access to such medium. The results showed that it is possible to measure glucose with carbon pencil leads under these conditions. Of all the tested brands, electrodes fabricated using Foray pencil leads presented a more stable response and more catalytic CVs despite producing smaller currents. Therefore, they were selected for integration in the microfluidic channels.

**Figure 6:**
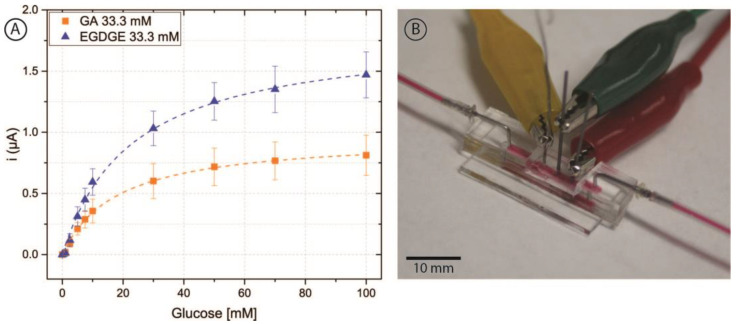
(**A**) Chronoamperometric calibration curves of 0–100 mM glucose in RPMI-1640 culture medium and 5% FBS employing pencil lead electrodes modified with OsBPEI/GOx/GA or OsBPEI/GOx/EGDGE in the microfluidic channel. Error bars represent the standard deviation of three different electrodes. Dashed lines represent the corresponding curves employing the K_m_^app^ and i_max_ calculated through non-linear fitting. (**B**) Photograph of the microfluidic channel with the integrated electrodes. Red dye has been injected in the channel to aid in its visualization.

Once glucose detection was demonstrated with carbon pencil leads, these modified electrodes were incorporated into the microfluidic channel (Figure 6B). As depicted in Figure 1, the electrodes protruded from the PDMS and into the channel approximately 597 ± 21 µm. For this brand of electrodes, microscopy measurements yielded a diameter of 555 ± 12 µm. Thus, the approximate geometric area exposed to the solution was 0.0132 ± 0.0005 cm^2^. To analyze the behavior of the hydrogels, 0–100 mM glucose calibration curves were performed in RPMI-1640 culture medium spiked with 5% FBS at a nominal flow rate of 100 μL/min (Figure 6A). These evaluations had a better performance than carbon SPEs evaluated off-chip, yielding increased stability and linear range with both cross-linkers.

These results show that pencil leads are suitable for integration of enzyme-modified electrodes into microfluidic chips. Experiments were performed under flow conditions and compositions representative of mammalian cell cultures. Although reports exist of the use of PGE for other complex biological matrices such as real samples (e.g., urine, plasma, and serum, Table S3) and defined media for microorganism culture, to the best of the authors’ knowledge there is no report of their use in media suitable for mammalian cell cultures. This is relevant as future work is directed towards the integration of these electrodes in organ-on-a-chip models. Besides being less prone to passivation and fouling, second generation enzymatic biosensors have the added benefit of being independent of the oxygen concentration in the medium. This is advantageous in cancer-on-a-chip platforms and other cell cultures performed in hypoxia. As well, H_2_O_2_ production is decreased in second-generation sensors, causing less cell damage than first-generation sensors. Although this problem can be somewhat ameliorated by including peroxidases in the sensor structure, this adds further complexity. The approach presented here is comparably simpler and suitable for further development and inclusion in mammalian cell culture chips.

The process of electrode integration in the chips used in this work is very similar to the integration of the fluidic connectors typically used in PDMS-based microfluidics. As such, scalability of this strategy is subject to the same advantages and limitations usually observed for PDMS-based devices. While soft lithography is a great technique for fast prototyping and fabrication of highly customized chips for specialized laboratory use, other fabrication techniques (e.g., lamination, hot embossing, and injection molding) are more suitable for scalability into mass manufacturing. Similarly, if high-throughput applications are intended where a large number (e.g., >100) of ports or sensors are required in a single device, the manual labor required in PDMS chips might not make it the best choice. Nevertheless, 3D cell culturing has been identified as one of the areas in which replica molding (by means of PDMS-based soft lithography) will likely still play a significant role in the near future because of the high biocompatibility of the materials and ease of custom microfabrication iterations for design improvement [65].

Future work will include the integration of these sensors in microfluidic chips containing cultured cells. Furthermore, an individual selectivity screening of each of the components of the medium can help us identify interferents and develop strategies to minimize their effects. While sensor reproducibility was acceptable, repeatability is currently limited by the sensor stability. Therefore, different approaches are being considered to improve stability and therefore overall sensor performance.

## 4. Conclusions

Biomedical scientists often think of microfluidic 3D cell culture platforms as a faster and more ethical way to study the interactions of substances with organs. These studies can greatly benefit from the inclusion of sensors within the channels to study the variations of the metabolites in the cell culture medium. These complex matrices, however, are known to present a challenge for some of the sensing platforms. In this work, hydrogels were prepared containing glucose oxidase, a cross-linker and branched polyethyleneimine (with or without Os-containing substituents). It was shown that carbon is a suitable material to immobilize such hydrogels and detect glucose. While in phosphate, buffer glutaraldehyde-based hydrogels presented better performance, EGDGE-based ones performed better in RPMI-1640 culture medium. The use of pencil lead electrodes was demonstrated as a simple and low-cost alternative to integrate carbon electrodes into microfluidic channels. The introduction of the hydrogel-modified electrodes did not damage the sensing layers, as evidenced by their satisfactory performance in flow-based calibration curves. These second-generation enzymatic biosensors presented a Michaelian response, while comparable first-generation sensors did not show a current dependence on the glucose concentration in cell culture medium. This is explained in terms of the different mechanisms of electron transfer from the enzyme to the electrode. Diffusion-based first-generation sensors are easily affected by substances blocking the path. Instead, hopping-based second-generation sensors make use of the existing network of redox moieties that persists even in the undesired presence of adsorbed or absorbed species. Therefore, the system presented here is a suitable candidate for incorporation in microfluidic mammalian cell cultures.

## Figures and Tables

**Table 1:** Comparison of limit of detection (LOD), limit of quantification (LOQ), sensitivity, stability, linear range, and coefficient of determination (R^2^) of different hydrogel compositions.

Hydrogel	LOD (mM)	LOQ (mM)	Sensitivity (µA/mM cm^2^)	Stability ^†^ (% j_max_)	Linear Range (mM)	R^2^
**Screen printed electrodes in 0.1 M pH 7.4 PB**						
**OsBPEI/GOx/GA** **3.33 mM**	0.48	1.59	3.36	7.59	0–5	0.997
**OsBPEI/GOx/GA** **17.5 mM**	0.89	2.98	3.82	9.14	0–7.5	0.994
**OsBPEI/GOx/GA** **33.3 mM**	0.12	0.39	4.64	19.5	0–5	0.987
**OsBPEI/GOx/EGDGE 3.33 mM**	0.09	0.30	2.90	1.33 *	0–7.5	0.998
**OsBPEI/GOx/EGDGE 17.5 mM**	0.42	1.40	1.74	1.34 *	0–7.5	0.980
**OsBPEI/GOx/EGDGE 33.3 mM**	0.61	2.05	2.66	7.23 *	0–5	0.993
**BPEI/GOx/GA** **33.3 mM**	3.05	10.2	2.96	100	0.5–5	0.994
**BPEI/GOx/EGDGE** **33.3 mM**	36.38	121.28	4.64	53.6	0.5–7.5	0.911
**Screen printed electrodes in RPMI-1640**						
**OsBPEI/GOx/GA** **33.3 mM**	1.41	4.71	5.22	12.2	0–5	0.970
**OsBPEI/GOx/EGDGE 33.3 mM**	0.28	0.93	9.04	50.7	0–5	0.995
**Pencil lead electrodes in RPMI-1640 with 5% FBS (on-chip)**						
**OsBPEI/GOx/GA** **33.3 mM**	1.49	4.97	3.10	52.9	0–7.5	0.989
**OsBPEI/GOx/EGDGE 33.3 mM**	0.50	1.67	4.69	49.9	0–10	0.993

^†^ Stability is reported as the remaining current expressed as percentage of the initial j_max_. * Data obtained after the second evaluation of the hydrogel.

## Data Availability

Not applicable.

## Supplementary Materials

The following supporting information can be downloaded at: https://www.mdpi.com/article/10.3390/bios13060582/s1, Figure S1. (A) Electrochemical cell setup with a carbon screen printed electrode (SPE.) (B) Carbon SPE with a carbon working (WE) and counter (CE) electrodes, and a C/Ag|AgCl mixture as reference electrode (RE).; Figure S2. Cyclic voltammograms of hydrogels containing glucose oxidase (GOx) cross-linked to branched polyethyleneimine modified with Os(bpy)_2_Cl(pyCOH) (OsBPEI) using either glutaraldehyde (GA) or ethylene glycol diglycidyl ether (EGDGE). (A) OsBPEI/GOx/GA and (B) OsBPEI/GOx/EGDGE hydrogels deposited on carbon SPEs in 0.1 M pH 7.4 phosphate buffer (PB) in the absence of glucose. Scan rate: 2 mV/s..; Figure S3. Dependence of the current of SPEs modified with OsBPEI/GOx/GA 33.3 mM (A,B) or OsBPEI/GOx/EGDGE composition (33.3 mM) (C,D) with respect to the potential scan rate (A,C) or square root of the scan rate (B,D) in 0.1 M pH 7.4 PBin the absence of glucose.; Table S1: Comparison of apparent Michaelis–Menten constant (K_m_^app^) and maximum current density (j_max_) of different hydrogels compositions. Data from three independent electrodes are presented, as well as their mean value.; Table S2. Comparison of K_m_^app^ and j_max_ of different hydrogels compositions. Data from three sequential calibration curves on a single electrode are presented.; Figure S4. Electrochemical calibration curves performed with carbon pencil leads of different brands onto which hydrogels were deposited using GA as cross-linker. Chronoamperometric signals were measured in 0.1 M pH 7.4 PB and the current at t = 2 min taken as analytical signal; Table S3. Comparison of analytical performance parameters of GOx based biosensors. References [66,67] are cited in the Supplementary Materials.
