# Supplementary material for: Development of a Redox-Polymer-Based Electrochemical Glucose Biosensor Suitable for Integration in Microfluidic 3D Cell Culture Systems
Source: Biosensors (Basel). 2023 May 27;13(6):582. doi: 10.3390/bios13060582 (PMC10295976; doi:10.3390/bios13060582)
Supplement: Supplementary file 1 [file biosensors-13-00582-s001.zip › biosensors-2323282-supplementary.pdf]

biosensors

MDPI

Supplementary Material

Development of a Redox-Polymer-Based Electrochemical Glucose Biosensor Suitable for Integration in Microfluidic 3D Cell Culture Systems

L. Navarro-Nateras, Jancarlo Diaz-Gonzalez, Diana Aguas-Chantes, Lucy L. Coria-Oriundo, Fernando Battaglini, José Luis Ventura-Gallegos, Alejandro Zentella-Dehesa, Goldie Oza, L. G. Arriaga and Jannu R. Casanova-Moreno.

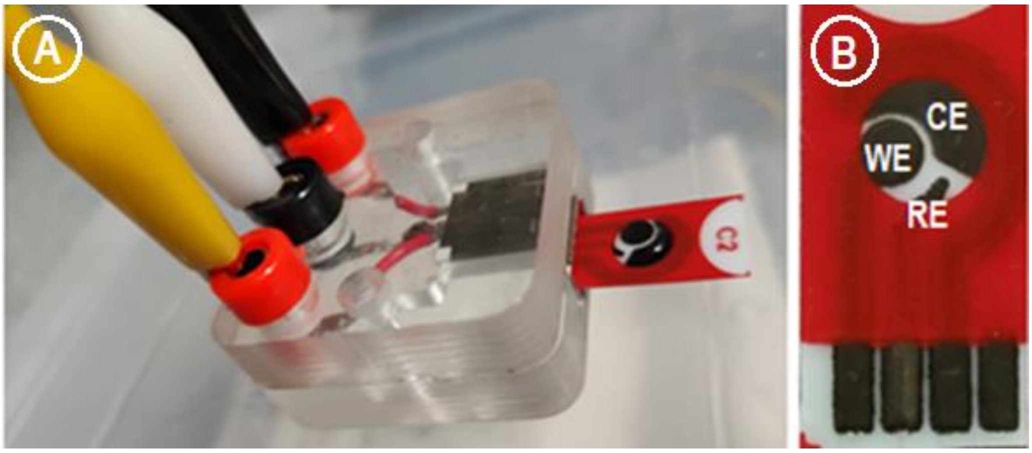

Figure S1. (A) Electrochemical cell setup with a carbon screen printed electrode (SPE.) (B) Carbon SPE with a carbon working (WE) and counter (CE) electrodes, and a C/Ag|AgCl mixture as reference electrode (RE).

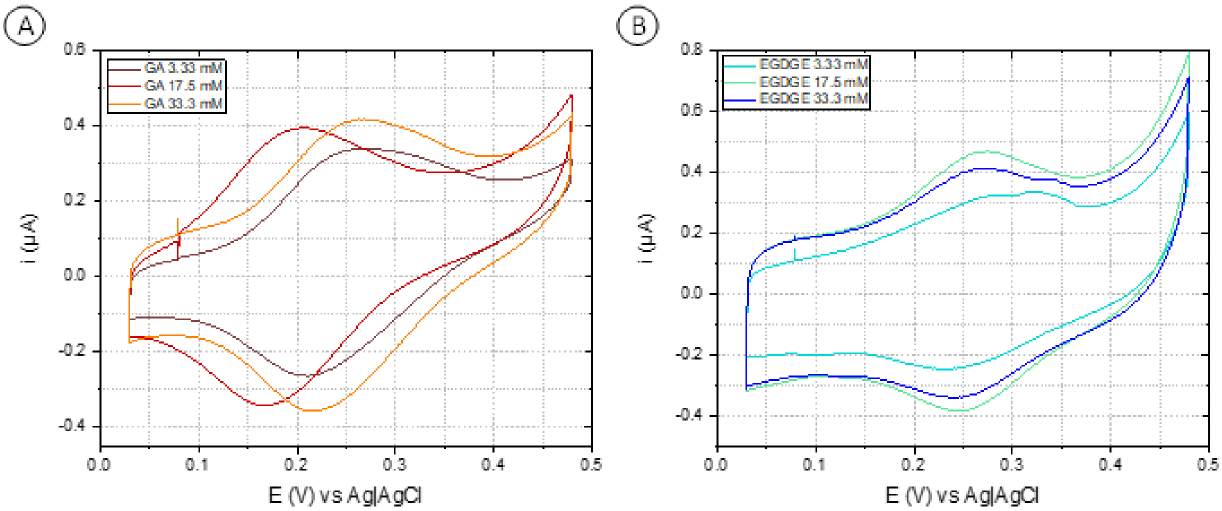

Figure S2. Cyclic voltammograms of hydrogels containing glucose oxidase (GOx) cross-linked to branched polyethyleneimine modified with $Os(bpy)_2Cl(pyCOH)$ (OsBPEI) using either glutaraldehyde (GA) or ethylene glycol diglycidyl ether (EGDGE). (A) OsBPEI/GOx/GA and (B) OsBPEI/GOx/EGDGE hydrogels deposited on carbon SPEs in 0.1 M pH 7.4 phosphate buffer (PB) in the absence of glucose. Scan rate: 2 mV/s.

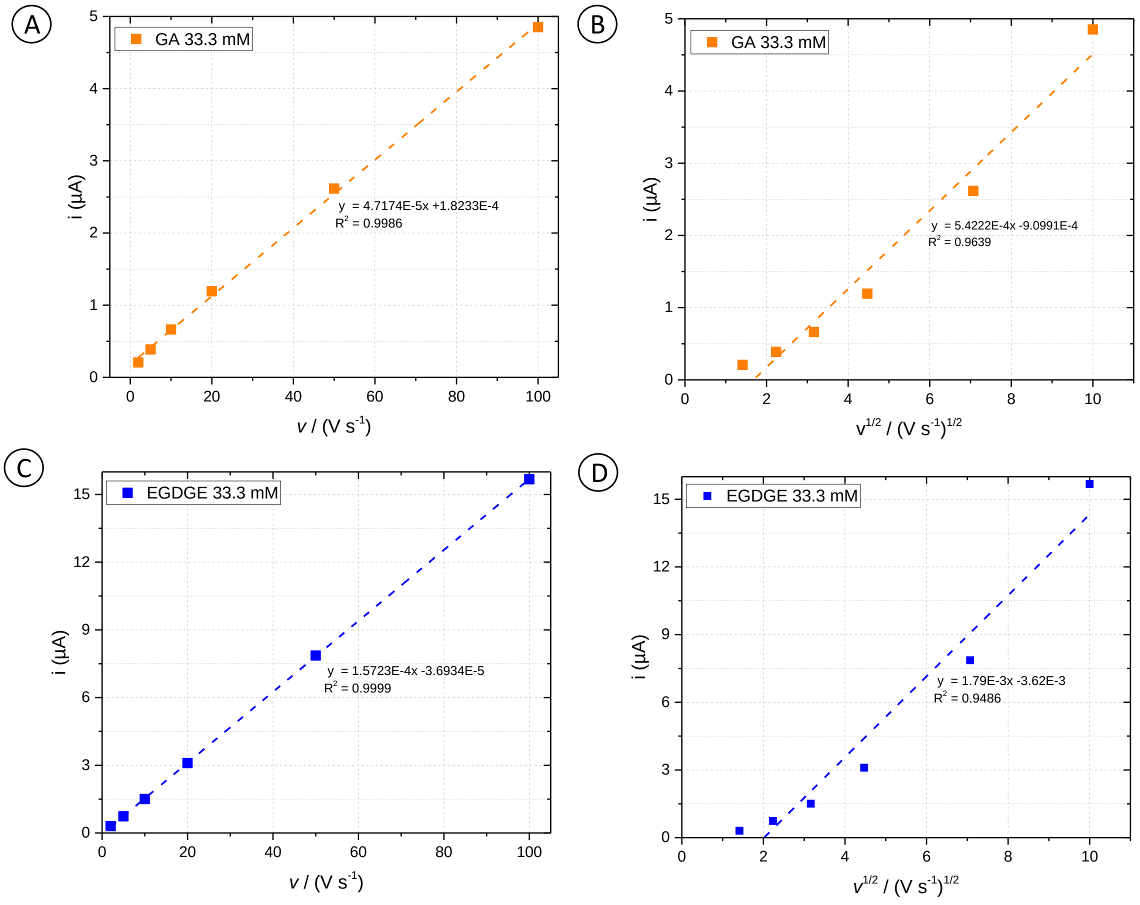

Figure S3. Dependence of the current of SPEs modified with OsBPEI/GOx/GA 33.3 mM (A,B) or OsBPEI/GOx/EGDGE composition (33.3 mM) (C,D) with respect to the potential scan rate (A,C) or square root of the scan rate (B,D) in 0.1 M pH 7.4 PB in the absence of glucose.

Table S1. Comparison of apparent Michaelis–Menten constant (K_m^{app}) and maximum current density (j_{max}) of different hydrogels compositions. Data from three independent electrodes are presented, as well as their mean value.

	K_m^{app} (mM)	j_{max} (µA/cm²)	K_m^{app} (mM)	j_{max} (µA/cm²)	K_m^{app} (mM)	j_{max} (µA/cm²)	K_m^{app} (mM)	j_{max} (µA/cm²)	R^2
							Mean		
SPEs in 0.1 M pH 7.4 PB									
OsBPEI/GOx/GA 3.33 mM	19.9	110	43.1	298	30.1	230	31.8±5.32	208±13.8	0.987
OsBPEI/GOx/GA 17.5 mM	6.16	58.4	10.6	45.1	14.4	89.0	10.1±0.75	63.5±1.27	0.995
OsBPEI/GOx/GA 33.3 mM	4.68	39.4	11.4	75.3	10.0	69.5	8.76±0.55	60.9±1.04	0.996
OsBPEI/GOx/EGDGE 3.33 mM	14.0	60.9	13.1	55.5	14.8	60.6	14.0±1.20	58.9±1.50	0.994
OsBPEI/GOx/EGDGE 17.5 mM	5.53	17.2	5.53	16.2	7.20	35.2	6.31±0.63	22.7±0.58	0.990
OsBPEI/GOx/EGDGE 33.3 mM	14.0	41.6	8.26	38.7	7.22	32.4	10.9±0.94	25.4±0.69	0.993
BPEI/GOx/GA 33.3 mM	5.61	37.5	6.30	80.3	5.55	63.5	5.89±0.75	60.4±1.85	0.982
BPEI/GOx/EGDGE	32.9	190	22.3	143.9	22.2	140	28.4±7.49	158±15.0	0.968

33.3 mM									
SPEs in Roswell Park Memorial Institute (RPMI-1640)									
OsBPEI/GOx/GA 33.3 mM	4.00	31.0	4.49	54.0	5.51	43.9	4.68±0.73	43.0±1.62	0.976
OsBPEI/GOx/EGDGE 33.3 mM	3.59	44.3	13.5	161	12.7	151	10.6±0.99	113±3.01	0.992
Carbon pencil leads in RPMI-1640 on-chip									
OsBPEI/GOx/GA 33.3 mM	15.2	86.2	23.1	58.2	18.5	74.8	18.0±1.13	72.6±14.3	0.997
OsBPEI/GOx/EGDGE 33.3 mM	20.4	147	22.8	141	26.3	121	22.8±1.87	136±39.3	0.996

Table S2. Comparison of K_m^{app} and j_{max} of different hydrogels compositions. Data from three sequential calibration curves on a single electrode are presented.

	K_m^{app} (mM)	j_{max} (μA/cm^2)	K_m^{app} (mM)	j_{max} (μA/cm^2)	K_m^{app} (mM)	j_{max} (μA/cm^2)
SPEs in 0.1 M pH 7.4 PB						
OsBPEI/GOx/GA 3.33 mM	19.9	110	12.0	24.5	7.14	8.35
OsBPEI/GOx/GA 17.5 mM	6.16	58.3	3.50	19.6	4.38	5.33
OsBPEI/GOx/GA 33.3 mM	2.34	22.0	1.99	10.5	2.23	4.29
OsBPEI/GOx/EGDGE 3.33 mM	14.0	60.9	5.01	0.81	-	-
OsBPEI/GOx/EGDGE 17.5 mM	5.53	17.1	3.30	0.23	-	-
OsBPEI/GOx/EGDGE 33.3 mM	14.0	41.6	20.8	3.01	-	-
BPEI/GOx/GA 33.3 mM	5.61	37.4	3.33	48.6	4.62	52.3
BPEI/GOx/EGDGE 33.3 mM	32.9	190	16.4	187	11.5	102
SPEs in RPMI-1640						
OsBPEI/GOx/GA 33.3 mM	4.49	54.0	1.89	9.74	1.31	6.61
OsBPEI/GOx/EGDGE 33.3 mM	3.59	44.3	9.48	26.2	10.4	22.5
Carbon pencil leads in RPMI-1640 on-chip						
OsBPEI/GOx/GA 33.3 mM	17.3	74.1	18.5	41.6	11.9	39.3
OsBPEI/GOx/EGDGE 33.3 mM	25.4	121	30.8	66.5	30.9	60.5

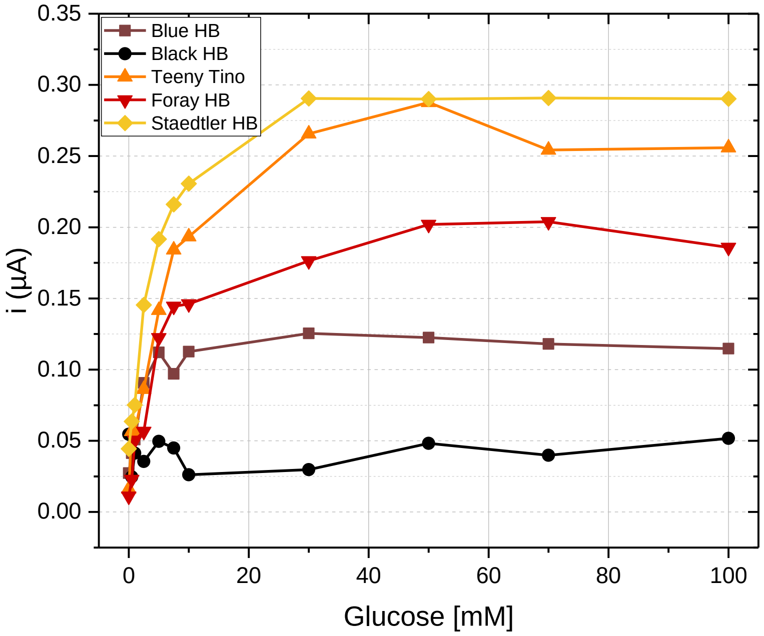

Figure S4. Electrochemical calibration curves performed with carbon pencil leads of different brands onto which hydrogels were deposited using GA as cross-linker. Chronoamperometric signals were measured in 0.1 M pH 7.4 PB and the current at t = 2 min taken as analytical signal.

Table S3. Comparison of analytical performance parameters of GOx based biosensors.

Composition	Electrode	LOD (mM)	LOQ (mM)	Linear range (mM)	Sensitivity µA mM^{-1} cm^{-2}	Matrix	Ref.
CB-PB-TEMPO-CNCs-GOx	SPE	0.0040	0.015	0.1–2	5.70	DMEM*/PB 1:4	[1]
m-PPD-pHEMA-GOx	Pt	0.0076	-	-	-	MEBM*	[2]
CNT-GR-GOx	GCE	0.0029	0.009	3–14	0.43	DMEM	[3]
Graphene-MWCNT/GOx/nafion	PGE	0.0149	-	0–39	35.2	PB	[4]
GO-GOx	PGE	0.0006	-	0.04–0.6	278.4	PB	[5]
ZnS-CdS-Chitosan-GOx	PGE	0.0030	-	0.01–1	11.5	BRBS*/KCl	[6]
OsBPEI-GOx-GA	PGE	1.4900	4.970	0–7.5	3.10	RPMI	This work
OsBPEI- GOx- EGDGE		0.5000	1.670	0–10	4.69		

* Dulbecco's Modified Eagle Medium (DMEM)
*Mammary epithelial basal medium (MEBM)
*Britton-Robinson buffer solution (BRBS)

1. Tang, Y.; Petropoulos, K.; Kurth, F.; Gao, H.; Migliorelli, D.; Guenat, O.; Generelli, S. Screen-Printed Glucose Sensors Modified with Cellulose Nanocrystals (CNCs) for Cell Culture Monitoring. *Biosensors* **2020**, *10*, 125. https://doi.org/10.3390/bios10090125.
2. Dornhof, J.; Kieninger, J.; Muralidharan, H.; Maurer, J.; Urban, G.A.; Weltin, A. Microfluidic organ-on-chip system for multi-analyte monitoring of metabolites in 3D cell cultures. *Lab Chip* **2022**, *22*, 225–239. https://doi.org/10.1039/d1lc00689d.
3. Madhurantakam, S.; Babu, J.K.; Balagruru Rayappan, J.B.; Maheswari Krishnan, U. Fabrication of mediator-free hybrid nano-interfaced electrochemical biosensor for monitoring cancer cell proliferation. *Biosens. Bioelectron.* **2017**, *207,* 832–841. https://doi.org/10.1016/j.bios.2016.09.039.
4. Torrinha, Á.; Montenegro, M.C.B.S.M.; Araújo, A.N. Microfluidic Platform with an Embedded Pencil Graphite Electrode Biosensor for the Detection of Glucose and Cadmium. *J. Electrochem. Soc.* **2019**, *166*, B155–B160. https://doi.org/10.1149/2.1221902jes.

5. Sehat, A.A.; Khodadadi, A.A.; Shemirani, F.; Mortazavi, Y. Fast immobilization of glucose oxidase on graphene oxide for highly sensitive glucose biosensor fabrication. *Int. J. Electrochem. Sci.* **2015**, *10*, 272–286.
6. Saglam, Ö.; KIzIlkaya, B.; Uysal, H.; Dilgin, Y. Biosensing of glucose in flow injection analysis system based on glucose oxidase-quantum dot modified pencil graphite electrode. *Talanta* **2016**, *147*, 315–321. https://doi.org/10.1016/j.talanta.2015.09.050.
